# Supplementary material for: ZnO Tips Dotted with Au Nanoparticles—Advanced SERS Determination of Trace Nicotine
Source: Biosensors (Basel). 2021 Nov 19;11(11):465. doi: 10.3390/bios11110465 (PMC8615426; doi:10.3390/bios11110465)
Supplement: Supplementary file 1 [file biosensors-11-00465-s001.zip › biosensors-1409452-supplementary.pdf]

ZnO Tips Dotted with Au Nanoparticles—Advanced SERS Determination of Trace Nicotine

Jiaying Cao, Yan Zhai, Wanxin Tang *, Xiaoyu Guo, Ying Wen and Haifeng Yang *

The Education Ministry Key Lab of Resource Chemistry, College of Chemistry and Materials Science,
Shanghai Normal University, Shanghai 200234, China; 1000479846@smail.shnu.edu.cn (J.C.); 1000459032@smail.shnu.edu.cn (Y.Z.); gxy2012@shnu.edu.cn (X.G.); ying.wen@shnu.edu.cn (Y.W.)
* Correspondence: wanxintang@shnu.edu.cn (W.T.); hfyang@shnu.edu.cn (H.Y.)

Calculation of the diameter of AuNPs, according to equation 1 [1]

$$d = \begin{cases} 3 + 7.5 \times 10^{-5} X^4, X \ll 23 \\ \left[\sqrt{X-17} \ - 1\right]/0.06, X \gg 23 \end{cases}, X = \lambda_{max} - 500$$

Where is the position of the surface plasmon resonance peak, d is the diameter of AuNPs.

Calculation of enhacement factor

The EF value can be estimated using the following equation 2 [2]:

$$EF = (I_{surface}/I_{solution}) \times (N_{solution}/N_{surface}) = \frac{I_{surface} N_{solution}}{I_{solution} N_{surface}}$$

In this equation, $I_{surface}$ stands for the signal intensities of the SERS substrate，and $I_{solution}$ stands for the the signal intensities of the reference solution. $N_{solution}$ and $N_{surface}$ are the numbers of molecules probed in a reference solution and on nanomaces SERS substrates, respectively. $I_{surface}$ and $I_{solution}$ can be obtained from the spectra directly while $N_{solution}$ and $N_{surface}$ need to be calculated on the basis of the estimation of the corresponding sample area. $N_{solution}$ and $N_{surface}$ can be obtained according to the reported method [3].

The characteristic band at 1363 cm^{-1} was chosen for the EF estimation. The SERS signal intensities ($I_{surface}$) were about 1785 for nanomace SERS substrates and the $I_{solution}$ were about 1079 in a reference solution [3].

Suppose the molecules uniformly dispersed on the substrate and then the density of the molecules on the substrate was assumed to be 1.0×10^{-11} mol/L $\times10$ μL×NA/0.126 cm^2(the

surface area of the substrate is ~12.6 mm²). The laser spot has a 1μm diameter and the surface area is about 7.9 ×10⁻⁹cm², so value is estimated as following:

$$N_{surface} = 1.0 \times 10^{-11} mol/L \times 10 \times 10^{-6} L \times N_A \times \frac{7.9 \times 10^{-9} cm^2}{0.126 cm^2} = 3.7$$

In comparison, Raman spectra of a 10^{-3} M R6G aqueous solution were measured applying a laser intensity of 1.7 mW. And the effective excitation volume in solution was estimated as ~400 μm³ [4] to give $N_{solution}$ ~ 2.4 ×10^{8} and a normal Raman intensity of ~4.5 × 10^{-6} counts per R6G molecule.

In this way, the EF of the substrate can be obtained as high as 1.07×10^{8}.

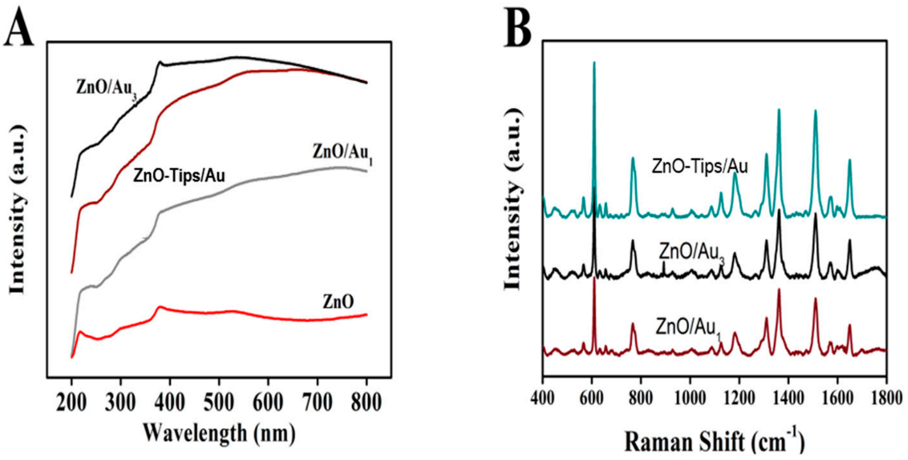

Figure S1. (A) UV-vis spectra of ZnO/Au substrates, prepared by adding 1, 2, and 2.5 mL of 1 % trisodium citrate solution, which denoted as ZnO/Au_1, ZnO-Tips/Au and ZnO/Au_3, respectively. (B) SERS spectra of R6G (10^{-7} mol/L) acquired on different ZnO/Au substrates.

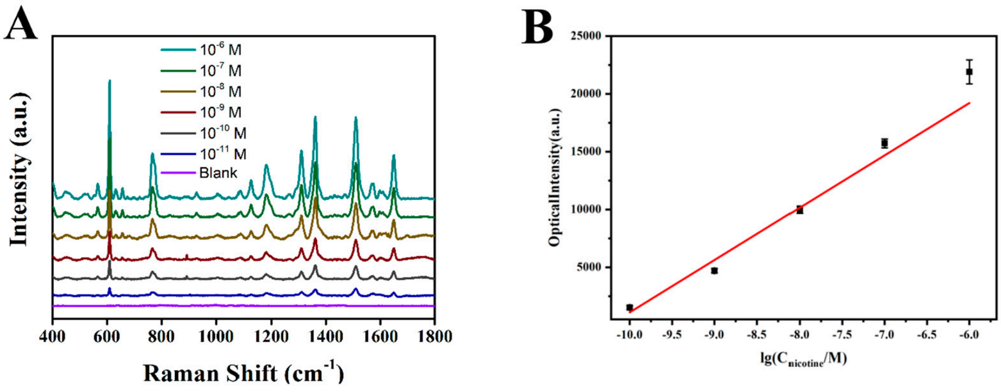

Figure S2. (A) SERS spectra of R6G solutions with different concentrations recorded on ZnO/Au. (B) Calibration plot based on Raman intensity at 1363 cm⁻¹ for R6G based on ZnO-Tips/Au substrate.

Table S1. Main SERS Band Assignments of Nicotine.

SERS/cm^{-1}	Assignment
679	Pyridine in-plane ring deformation
1031	Pyridine symmetric ring "breathing"
1056	Pyridine trigonal ring "breathing"
1194	Pyridine in-plane C-H deformation
1589	Pyridine ring stretch

References

1. Baptista, P.; Pereira, E.; Eaton, P.; Doria, G.; Miranda, A.; Gomes, I.; Quaresma, P.; Franco, R., Gold nanoparticles for the development of clinical diagnosis methods. *Anal Bioanal Chem* 2008, *391*, 943-50.

2. Xiao, C.-h.; Xiao, B.-x.; Wang, Y.-d.; Zhang, J.; Wang, S.-m.; Wang, P.; Yang, T.-y.; Zhao, R.; Yu, H.; Li, Z.-f.; Zhang, M.-z., Synthesis of ZnO nanosheets decorated with Au nanoparticles and its application in recyclable 3D surface-enhanced Raman scattering substrates. *RSC Advances* 2015, *5*, 17945-17952.

3. Huang J, Chen F, Zhang Q, et al. 3D silver nanoparticles decorated zinc oxide/silicon heterostructured nanomace arrays as high-performance surface-enhanced Raman scattering substrates[J]. *ACS applied materials & interfaces*, 2015, *7*, 5725-5735.

4. Cai W B, Ren B, Li X Q, et al. Investigation of surface-enhanced Raman scattering from platinum electrodes using a confocal Raman microscope: dependence of surface roughening pretreatment[J]. *Surface Science*, 1998, *406*, 9-22.
